# Effects of Different Processing Methods and Internal Components on Physicochemical Properties and Glycemic Index of Adzuki Bean Powder

**DOI:** 10.3390/foods10081685

**Published:** 2021-07-21

**Authors:** Feiyue Ren, Xiaoxue Yang, Lili Wang, Sumei Zhou

**Affiliations:** 1 Beijing Advanced Innovation Center for Food Nutrition and Human Health, Beijing Engineering and Technology Research Center of Food Additives, School of Food and Health, Beijing Technology & Business University (BTBU), Beijing 100048, China; 20200101@btbu.edu.cn; 2 Institute of Food Science and Technology, Chinese Academy of Agricultural Sciences, Beijing 100193, China; jyyw@caas.cn

**Keywords:** adzuki bean powder, estimated glycemic index (eGI), in vitro digestibility, steamed cooking, enzymatic processing

## Abstract

The estimated glycemic index (eGI) value of adzuki bean powder prepared by steamed cooking (SC), extruded cooking (EC) and roller cooking (RC) was studied comparatively. Results showed that RC had the highest eGI, with 80.1, and both EC and SC resulted in a lower eGI value of 70.0 and 49.7, respectively. Compared with the EC and RC methods, the SC method provided a more intact physical barrier for starch digestion, resulting in a less destroyed cell structure. As the essential components that form the cell wall, the study further investigated the effects of protein and fiber on physicochemical properties, in vitro starch digestibility and the eGI of adzuki bean powder processed with the SC method. Viscozyme and Protamax were used to obtain the deprotein and defiber samples. Results showed that the SC treatment with Viscozyme and Protamax, respectively, had significant effects on in vitro starch digestibility. The eGI of different samples were given as follows: steamed cooking adzuki bean powder (49.7) < deproteined adzuki bean powder (60.5) < defibered adzuki bean powder (83.1), which indicates that fiber may have a greater influence on the eGI than protein.

## 1. Introduction

The adzuki bean (*Vigna angularis*) is widely consumed in many countries and was mainly cultivated in China, North Korea, Japan and other southeast Asian countries. It is rich in protein, starch, dietary fiber, phytochemicals and other nutrients [1,2]. Regular consumption of the adzuki bean can improve the metabolic function of the human body, and especially the glucose and lipid metabolism of diabetic patients, which is beneficial to their health [1,2,3,4]. The adzuki bean has been processed and consumed in different forms for a better taste, such as adzuki bean powder and paste [5,6,7].

The structure and digestibility of starchy food can be affected by different processing methods, such as milling (i.e., wet and dry milling) and cooking (e.g., steaming, extrusion), and could further influence the glycemic response [8,9,10]. The use of different milling methods could result in a different content of damaged starch. Compared with dry milling, wet milling contributes to the integrity of the starch granules, leading to a lower level of starch digestibility and lower eGI value [11]. Regarding different cooking techniques for the processing, steamed cooking uses moist heat as the medium and extrusion processing uses thermal–shear action [12,13]. However, few studies have been carried out in order to compare different milling and cooking processing methods with regard to the digestibility and eGI of adzuki beans. Moreover, as eGI values can be affected by physicochemical structures [14,15], it is worth studying the structural properties of adzuki bean powder with different processing methods.

Epidemiological and clinical studies reported that whole cereals and pulses have relatively low glycemic index (GI), which could better reduce the risk of chronic diseases, such as obesity and type 2 diabetes [6]. It is evident that protein plays a vital role in reducing GI in cereals, due to the fact that protein has an embedding effect on starch, which limits the contact between amylase and starch, making it difficult to digest, and hence reducing the glucose response [16,17]. Other studies have also shown that the cell walls of beans could limit both the starch absorption of water and the gelatinization process [16,17].

The interaction of starch, protein and fiber on the nutritional and functional properties of cereal has been investigated by a number of researchers [18,19,20,21]. It has been proven that the starch–protein–fiber interaction plays a vital role in obtaining the hypoglycemic property of cereals. Studies have shown that protein and fiber encapsulating starch granules reduce the overall in vitro starch digestibility [21,22,23]. However, little is known on the effect of fiber and protein on the in vitro starch digestibility and glycemic index of adzuki bean powder.

This study aims to investigate the effects of different processing methods: steamed cooking, roller cooking and extruded cooking on the eGI of adzuki bean powder. Selecting the treatment with the best result in eGI, the present study further examines the impact of fiber and protein on eGI.

By studying the gelatinization characteristics and structural properties of adzuki bean powder with different processing methods, the findings will optimize the existing cooking methods of adzuki bean powder with a lower eGI value. More significantly, the findings will provide more in-depth scientific information on the effect of fiber and protein on eGI in order to develop healthier adzuki bean foods.

## 2. Materials and Methods

### 2.1. Materials

Adzuki beans (Jihong 0015) were kindly provided by the Institute of Crop Germplasm Resources (CAAS), Beijing, China. Total starch kit, Glucose Oxidase/peroxidase (GOPOD) kit, Amyloglucosidase (AMG, 3260 U/mL), pepsin (3200 U/mg) and α-amylase (10 U/mg) were purchased from Megazyme (Bray Co., Wicklow, Ireland). Porcine pancreas pancreatin (A1750, 4 × USP activity), fluorescein isothiocyanate isomer I (FITC, F7250, Sigma) and calcofluor-white stain (Sigma, 18909) were purchased from Sigma-Aldrich (St. Louis, MO, USA). Viscozyme and Protamax were kindly provided by Novozymes (Bagsværd, Denmark). All other chemical reagents used in this study were of analytical grade.

### 2.2. Preparation of Adzuki Bean Powder with Different Methods

Steamed cooking (SC): adzuki beans (100 g) were soaked in water overnight (12 h) with a ratio of adzuki beans to water of 1:3 and then cooked at 100 °C for 40 min. When they were cooled down, the materials were beaten into paste in a high-speed stirrer, ground by colloidal grinding and passed through a 60-mesh sieve. The wet adzuki bean powder was freeze-dried (i.e., steam cooked powder, SC) for subsequent analysis.

Roller cooking (RC): adzuki beans (100 g) were milled with cyclone (CT410, FOSS, Suzhou, China) in order to pass through a 60-mesh sieve, then the slurry was prepared with a ratio of raw adzuki bean powder to water of 1:2. The slurry was ground finely by colloidal grinding. The materials were poured on the surface of the drum dryer with a temperature of 150 °C, rotational speed at 1350 rmp and pressure at 0.3–0.8 Mpa. The collected materials were crushed through 60-mesh sieve, which was named as RC.

Extrusion cooking (EC): adzuki beans (100 g) were milled through 60-mesh sieve, the moisture content was adjusted to 40%, and then the beans were extruded through the twin screw extrusion system (Brabender, Germany); extrusion machine barrel temperature program: 50 °C, −60 °C, −80 °C, −100 °C, −120 °C, screw speed: 160 r/min. The extrudates were collected and placed in an oven at 40 °C, drying for 12 h. After drying and grinding (60-mesh), the extrudates were sealed and stored for subsequent analysis.

Raw adzuki bean powder (R): this was obtained by taking 100 g of adzuki bean and grinding it through a 60-mesh sieve, which was named as R, and taken as the control samples of the above cooking samples.

### 2.3. Treatment of Adzuki Bean Powder with Viscozyme and Protamax

Control sample (SC): the adzuki bean powder prepared with steamed cooking was taken as control sample. The preparation method was as above.

Defiber powder (SC-DF): 100 g adzuki bean powder was soaked in water at 25 °C for 4 h with a ratio of adzuki beans to water of 1:3, cooked at 100 °C for 40 min, cooled down and then ground through colloidal grinding. Its pH was adjusted to 5.5, Viscozyme was added (0.5%, in the ratio of enzyme to substrate), it underwent hydrolysis at 45 °C for 2 h and was passed through a 60-mesh sieve. The wet adzuki bean powder was freeze-dried for further analysis. Similar studies with the Viscozyme treatment were used by Figueiredo, Yamashita, Vanzela, Ida and Kurozawa [22].

Deprotein powder (SC-DP): 100 g adzuki beans were soaked in water at 25 °C for 4 h with a ratio of adzuki beans to water of 1:3, cooked at 100 °C for 40 min, cooled down and then ground through colloidal grinding. The pH of the resulting powder was adjusted to 7.0, complex Protamax was added (addition amount: 1.0%, in the ratio of enzyme to substrate), it underwent hydrolysis at 50 °C for 2 h and was then passed through a 60-mesh sieve. The wet adzuki bean powder was freeze-dried for further analysis. Similar studies with the Protamax treatment were used by Annor, Marcone, Bertoft and Seetharaman [18].

### 2.4. Damaged Starch Content

The damaged starch content was measured using the AACC approved method 76–31.01 [24,25]. The damaged starch content of adzuki bean powder was determined by enzyme colorimetry (Megazyme damaged starch kit)

### 2.5. In Vitro Digestibility

In vitro digestion was carried out using porcine pancreatic α-amylase and AMG with the method described by Sopade and Gidley [26] with slight modifications. Raw adzuki bean powder was then cooked for 20 min until fully gelatinized. The fully gelatinized adzuki bean powder was the control sample (C). The samples (50 mg, dry weight basis) were suspended in 2 mL of distilled water and 20 mL of sodium acetate buffer (0.2 M, pH 6.0, containing 0.49 mM MgCl_2_, 200 mM CaCl_2_) and then equilibrated at 37 °C for 15 min. Three glass balls (10 mm diameter) and 5 mL of the enzyme solution (containing 5 mg of pancreatin and 50 μL of AMG in sodium acetate buffer solution) were added to each tube. The mixture was incubated in a water bath at 37 °C with stirring at 300 rpm. The digesta (0.5 mL) were collected at 5, 15, 30, 45, 60, 90, 120 and 180 min, and the digestion was stopped by adding 0.5 mL of 95% ethanol. The digestibility of gelatinized sample was determined from the amount of glucose released in the supernatant, tested using the Megazyme glucose kit, with a conversion factor of 0.9 (the ratio of the molecular weight of the hydroglucose monomer unit in starch to that of glucose).

The digestion kinetic profiles were then fitted to the first-order equation to obtain the apparent digestion rate coefficient (k, min^−1^) [27,28]

C_t_ = C_∞_ (1 − e^−kt^) (1)

where C_t_, C_∞_ and k were the concentration at incubation time t, the equilibrium concentration and the kinetic constant, respectively. Using the hydrolysis curve (0–180 min), the hydrolysis index (HI) was calculated as the percentage of total glucose released from the samples [25]. The glycemic indices of the samples were estimated according to the equation [29]:

GI = 39.71 + 0.549HI(2)

### 2.6. X-ray Diffraction (XRD) and Relative Crystallinity

The X-ray diffraction and degree of crystallinity of adzuki bean powder were recorded using a powder X-ray diffractometer (Bruker D8 ADVANCE, Berlin, Germany) with Cu-Kαvalue of 1.54 radiation at room temperature. It was scanned at a rate of 2°/min from the diffraction angle (2θ) between 4° and 40°, using a voltage of 40 kV and filament current 30 mA. The crystallinity was calculated according to the equation below:

X_c_ = A_c_/(A_a_ + A_c_) (3)

where X_c_ is the crystallinity, A_c_ is the crystalline area and A_a_ is the amorphous area on the X-ray diffractogram.

### 2.7. Differential Scanning Calorimetry (DSC)

The thermal properties of each sample were examined using differential scanning calorimetry (DSC-Q200, New Castle, DE, USA). Approximately 3 mg sample was mixed with 6 mg deionized water and hermetically sealed in an aluminum pan, allowed to equilibrate for 12 h at room temperature and then scanned at a heating rate of 5 °C/min from 40 to 120 °C. The differential scanning calorimetry analyzer was calibrated using indium as a standard and an empty aluminum pan was used as the reference. The onset temperature (T_o_), peak temperature (T_p_) and enthalpy of gelatinization (ΔH) were calculated automatically.

### 2.8. Microscopic Observations

Morphology of the samples was studied by scanning electron microscope (SEM, Su8010, Hitachi, Japan) with 15 kV potential accelerator. For SEM, the adzuki bean powder was directly applied to a circular metal stub covered with double-sided adhesive carbon tape, then coated with platinum by a sputter coater. An accelerating voltage of 5 kV was used during imaging. For polarized pictures, a Motic BA310POL microscope (Motic Group Co., Ltd., Guangzhou, China) equipped with a cross polarizer was used.

### 2.9. Confocal Laser Scanning Microscopy

Confocal laser scanning microscopy (CLSM) was carried out following the method described by Zhang et al. [30] with slight modifications. Cell wall component and free or entrapped starch granules before and after enzymatic hydrolysis were observed by a double staining protocol employing fluorescein isothiocyanate (FITC) (specific to starch) and calcofluor-white (specific to cell walls) using a confocal microscope (LSM 700, Carl Zeiss, Germany) at 488 nm (Emmax 525 nm) and 405 nm (Emmax 433 nm) excitation wavelength, respectively [31]. Control and digested fractions were labelled at room temperature overnight using FITC, centrifuged at 3000 rpm for 60 s and then rinsed with Milli-Q water six times to remove the excess dye. The mixture was spread onto a glass slide and observed using a confocal laser scanning microscope (TCS-SP5, Leica, Germany) at 40 × 1.25 oil magnification.

### 2.10. Statistical Analysis

All the experiments were performed in triplicate and the experimental data were analyzed using analysis of variance (ANOVA), which were expressed as mean value ± standard deviation. A Duncan’s multiple range test was conducted in order to assess the level of significant differences among experimental mean values (*p* < 0.05). All statistical computations and analyses were conducted using SPSS version 13.0 (SPSS Inc., Chicago, IL, USA) for Windows.

## 3. Results and Discussion

### 3.1. In Vitro Digestion Characteristics of Adzuki Bean Powder under Different Processing Methods

**Figure 1:**
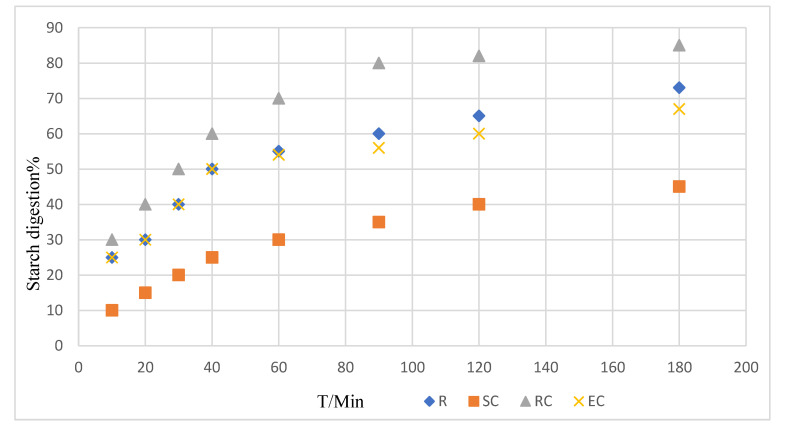
Digestion process of adzuki bean powder prepared by different processing methods. R: raw adzuki bean powder, SC: steamed cooking adzuki bean powder, RC: roller cooking adzuki bean powder, EC: extruded cooking adzuki bean powder.

**Figure 2:**
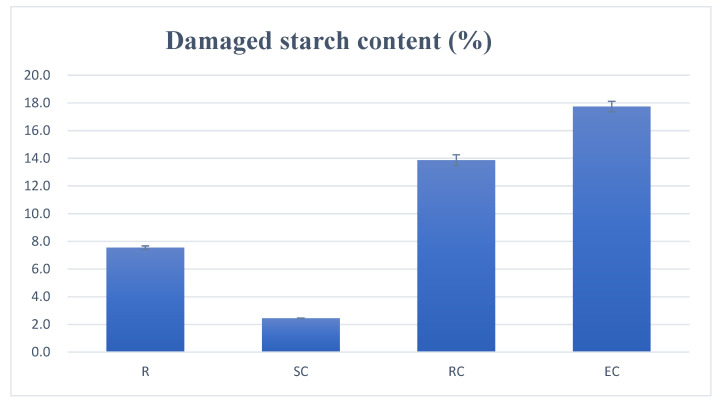
Damaged starch content of adzuki bean powder prepared by different processing methods.

Figure 1 displays the digestive kinetics curve of adzuki bean powder with different cooking methods. It shows that the SC and EC methods have relatively lower digestion rates compared with the RC. As shown in Table 1, cooking methods with the lowest eGI to the highest were: steamed cooking (SC, 49.7), extrude cooking (EC, 70.0) and roller cooking (RC, 80.1). The results showed that the final starch digestibility and eGI value of the SC samples decreased significantly compared with uncooked adzuki bean powder samples (R, 79.8). SC had a low content of damaged starch (2.44%, as shown in Figure 2) so the starch granules were almost intact, which required more heat to gelatinize the starch, whereas the EC (17.74%) and RC (13.86%) had a higher content of damaged starch, which may result in easier digestion. Compared with SC followed by wet milling, more mechanical and thermal energy were produced by EC and RC with the initial use of the dry milling for the raw bean flour, resulting in higher levels of damaged starch content [32]. The result has been supported by Dhital et al. [33], suggesting that during cooking processing, the cellular integrity of pulses remained and most of the starch granules retained an ordered structure, which could slow down the digestion rate to a great extent.

In addition, the gelatinization degree of starch granules and the physical barrier and other substances could be the factors affecting the enzymatic hydrolysis or digestion rate of starch. The SC method may prevent the swelling from water absorption and gelatinization of the starch granules during the cooking, restricting the digestion rate and eGI [34].

Different processing methods applied in the present study resulted in differences in eGI values. The impact of thermal properties, microstructure and crystallization characteristics of adzuki bean powder on eGI was further discussed.

### 3.2. Microstructure Analysis of Adzuki Bean Powder under Different Processing Methods

**Figure 3:**
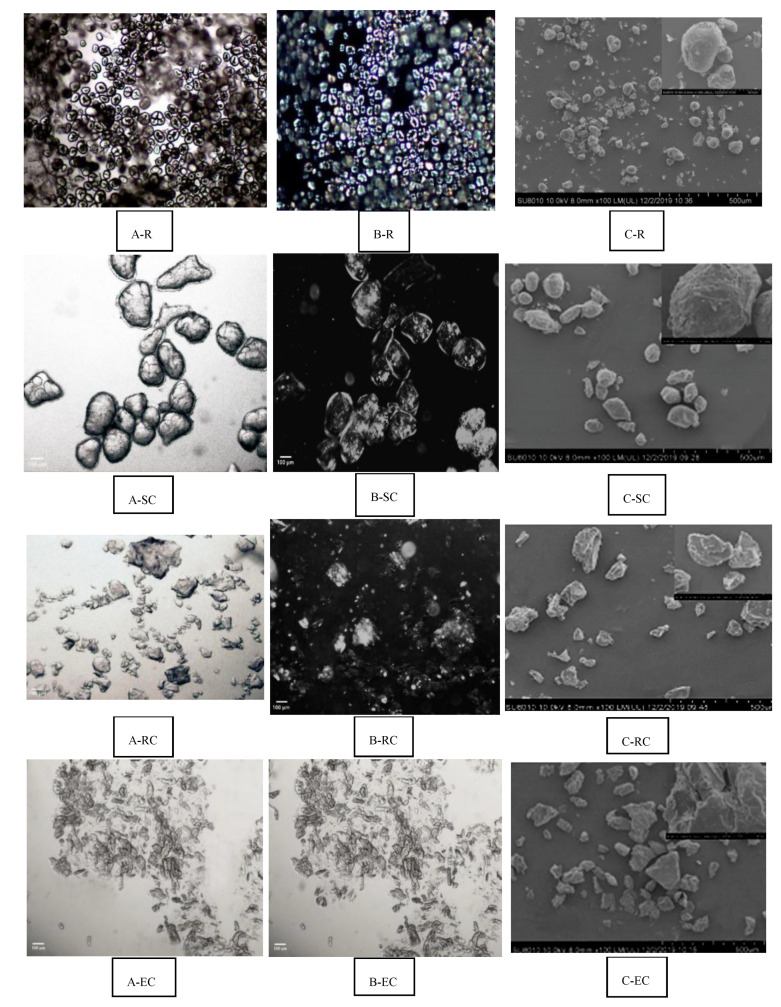
(**A**) polarized light, (**B**) bright field, (**C**) scanning electron micrographs showing adzuki bean powder with different processing methods. R: raw adzuki bean powder, SC: steamed cooking adzuki bean powder, RC: roller cooking adzuki bean powder, EC: extruded cooking adzuki bean powder.

The polarized light microscopical observations of raw and different processed adzuki bean powder are shown in Figure 3A,B and the scanning electron microscope (SEM) observation results are shown in Figure 3C. Based on Figure 3, steamed cooking (SC) maintained the integrity of the granules of adzuki bean powder, largely due to the wet milling processing. With the EC and RC methods, the adzuki bean powder samples were initially ground using the dry milling method, followed by extruding/rolling and freeze-drying. During the dry milling process, the starch granules of adzuki bean powder were damaged. The raw adzuki bean starch granules were birefringent with distinct Maltese crosses; however, the birefringence and Maltese crosses disappeared with different processed sample starch granules. Wang et al. [5] also found that both the degree of disappearance of the Maltese crosses under polarized light microscopy and the observed transformation of adzuki bean starch from ellipsoid to pie shape under SEM could reflect the degree of gelatinization.

Extruded adzuki bean powder was flat, round and had a long strip shape with a relatively dense structure. By contrast, the adzuki bean powder processed with the RC method showed an irregular thin slice and had almost no polarized structure. Roller-cooked adzuki bean powder had a looser binding between substances, and the starch was easier to contact with enzymes, whereas extruded adzuki bean powder was more compact, the substances were aggregated and combined and the contact between the enzymes and starch was less, leading to different eGI values.

The transparent cell wall structure on the outside of the granules can be observed when the adzuki bean powder was steam cooked, which was observed under the electron microscope. It was shown that only some polarized images of the cells were captured, indicating that the gelatinization of the sample was complete. The raw material of the SC processing method was whole beans, which maintained the integrity of the cell structure before cooking. According to the study of Dhital et al. [33], cell walls can still be observed after the whole cells are heated, and there is a certain physical barrier to amylase. Therefore, it is speculated that the cooking of the whole beans maintained the integrity of the cells to a great extent and hence reduced the eGI value.

### 3.3. Thermal Properties and Crystallinity of Adzuki Bean Powder under Different Processing Methods

The DSC results of adzuki bean powder after different processing methods are shown in Table 2. In the control group (R), the T_o_, T_p_ and ΔH were 65.98 °C and 73.87 °C, while 2.91 J/g and ΔH decreased in SC, RC and EC, but no significant difference was found in ΔH among the three processing methods. Figure 3 shows that different processing methods had a significant effect on the damaged starch contents of the adzuki bean powder. The results showed that the damaged starch content of the EC was the highest, while the SC had the lowest damaged starch content and intact starch granules exhibited the highest T_o_ value. All three processing methods led to starch gelatinization, but there was still disparity in eGI value. The reason could be attributed to the structural integrity, less damaged starch and particle integrity of adzuki bean powder.

Regarding microscopical observations, in SC adzuki bean powder, the starch was gelatinized. No gelatinization peak can be detected in the range of 25~100 °C for RC and EC adzuki bean powder, and no Maltese crosses can be observed under polarized light microscopy, which indicated that the starch has been completely gelatinized. The results demonstrated that in RC and EC, starch could better contact with water when the adzuki bean was ground into powder and cooked under the condition of sufficient water, thus reaching complete gelatinization. This means that during cooking, the water content could impact the gelatinization degree of starch and the eGI value of adzuki bean powder. In addition, compared to RC and EC, steam cooked processing (SC) with wet milling resulted in a lower content of damaged starch; the integrity of the starch granules remained and the structure of the starch in adzuki bean powder was relatively intact.

Moreover, starch relative crystallinity is an important parameter to characterize the crystallization properties of starch granules. With different processing methods (i.e., SC, RC and EC), the relative crystallinity of the adzuki bean powder starch decreased from 20.2% (R) to 17.13% (SC), 12.27% (RC) and 10.23% (EC), respectively. This explains that the processing methods can partially destroy the ordered structure of starch crystallinity [34,35,36]. The variation of relative crystallinity was small after SC treatment. It was speculated that the gelatinization of starch granules was more restricted by the cell wall and other components of the cell, and so the change in the ordered structure of starch granules was limited.

### 3.4. Comparison of Starch Crystal Structure of Adzuki Bean Powder under Different Processing Methods

**Figure 4:**
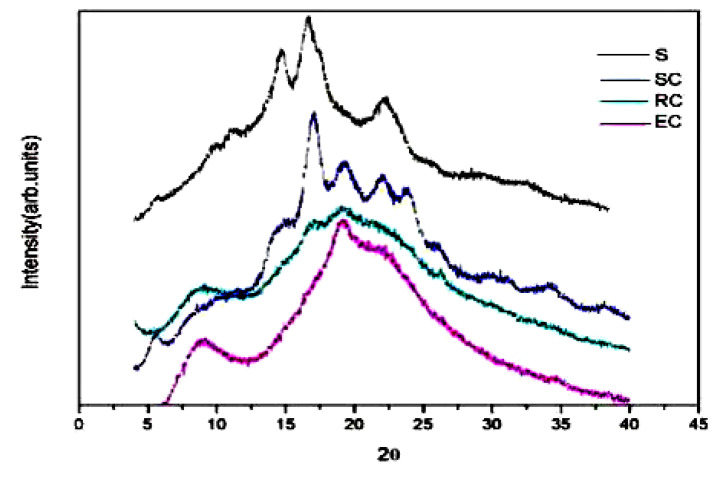
X-ray diffractograms of adzuki bean powder prepared by different processing methods.

The X-ray diffraction patterns of raw adzuki bean starch (R) and processed adzuki bean powder are presented in Figure 4. Figure 4 shows that the raw adzuki bean starch at 5.7°, 15.0°, 17.3° and 23.0°(2θ) has a strong peak, indicating the typical C_B_ type XRD curve.

Roller-cooked adzuki bean powder (RC) and extruded adzuki bean powder (EC) showed no obvious crystallization peak; the starch was fully gelatinized and the result was consistent with the result of DSC. For the steam-cooked adzuki bean powder, there was no starch crystallization peak at 15.0°, but a single peak at 17.3° and a double peak at 23.0°, indicating that the crystal structure was less damaged. SC was cooked with whole beans, where the intact cell wall structure can limit cell expansion. The starch structure cannot be fully extended due to the inclusion of the cell wall, so as to retain the ordered structure of starch granules and keep the integrity of the crystalline structure.

### 3.5. Microstructure of Deproteined and Defibered Adzuki Bean Powder under Steamed Cooking

Based on the above findings, we found that the SC method maintained the integrity of the cells with less damage and that the SC method in particular showed the lowest damaged starch content and eGI value. It is also well known that the steamed cooking treatment has a great influence on vegetable cell structure and texture [37]. Therefore, the following experiments were conducted with the samples produced with the SC method in order to further study the possible effects of the internal components (fiber and protein) in the outer cell wall of granules on estimating the glycemic index of adzuki beans during in vitro digestion.

**Figure 5:**
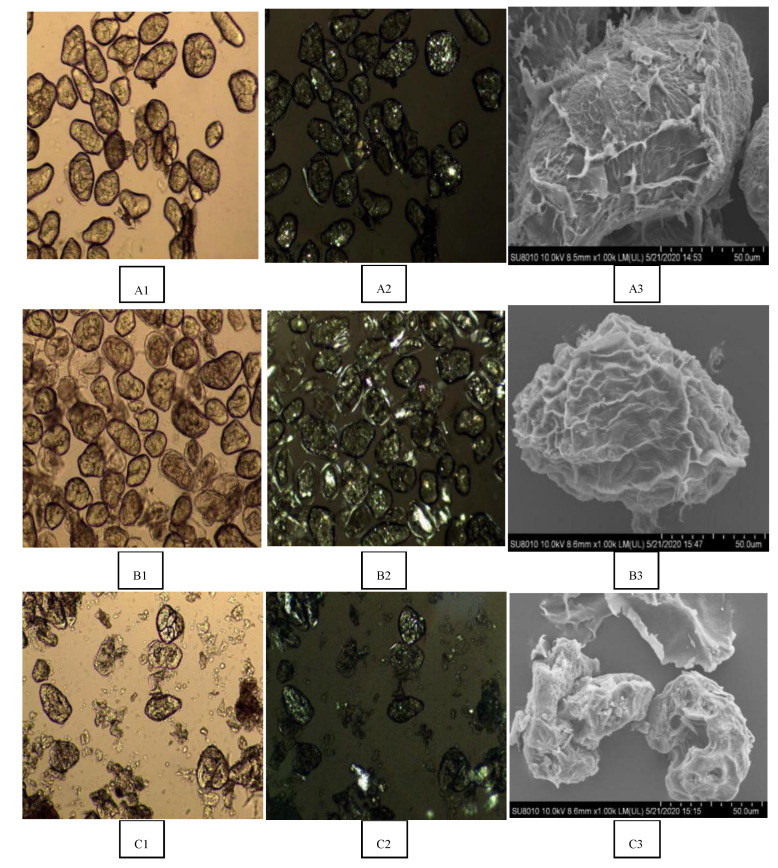
(**A**) polarized light, (**B**) bright field, (**C**) scanning electron micrographs showing the structural features of control (**A1**–**A3**) vs. deprotein (**B1**–**B3**) and defiber (**C1**–**C3**) granules of adzuki bean powder.

Light microscope images of adzuki bean powder treated with steam cooking with Protamax and Viscozyme are shown in Figure 5A,B. Protamax and Viscozyme were used to effectively degrade the protein and cell wall fibrous tissue and increase the release of cellular contents. A scanning electron microscope (SEM) of steam-cooked adzuki bean powders with Protamax (deprotein) and Viscozyme (defiber) are shown in Figure 5C.

After steamed cooking treatment with the complex Viscozyme (SC-DF), a scanning electron microscope showed that the cell structure of steam-cooked adzuki bean powders (SC-DF) was obviously deformed, and that the starch particles wrapped by the cell wall were gradually exposed [9]. Shrunken shells and a porous structure with a dent were observed as a result of the denaturation and aggregation of the cell membranes. This may be caused by the partial loss of the barrier properties of the adzuki bean powder cells due to steamed cooking with wet milling or a different enzyme.

Birefringent with Maltese crosses were observed in all cell samples under the polarized light microscopy. In Figure 5A,B, the surface of the adzuki bean powder cells showed some shrinkage, the birefringence of the adzuki bean powder cells under the polarized light field decreased slightly and the cell surface did not change significantly.

The scanning electron microscope showed that the granules of defibered adzuki bean powder adhered to each other, that the surface was rough and that the particle shape was irregular. A few fragments of cell walls around the granules could be observed under the light microscope. Large granules of the defibered adzuki bean powder disappeared.

The permeability of the adzuki bean powder cell walls may limit the ability of Viscozyme to pass through the cell wall to the inside of the cells. The cell wall permeability of adzuki bean powder cells can be damaged irreversibly by steamed treatment. In addition, the strength, porosity and stiffness of the cell wall can also be disrupted after thermal treatment, enabling Protamax and Viscozyme to pass through [6], leading to a higher in vitro digestion rate and eGI values in adzuki bean powder.

### 3.6. Localization of FITC Labelled Protamax and Viscozyme on Steamed Cooking Adzuki Powder

**Figure 6:**
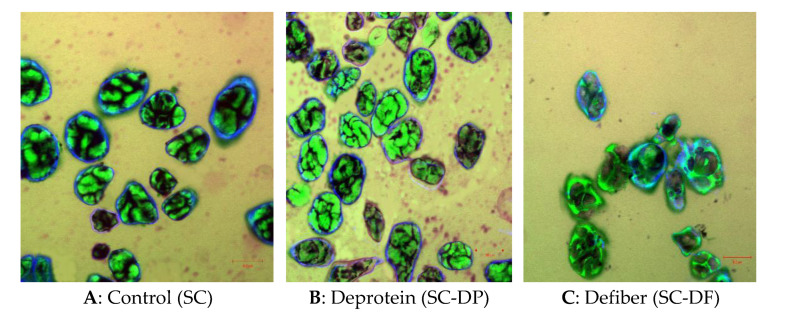
Schematic diagram showing isolated intact granules of steamed cooking adzuki bean powder (**A**), and broken granules (**B**: deprotein and **C**: defiber) of adzuki bean powder. FITC (green) and calcofluor-white (blue) labelling revealed the packing of intact granules and broken granules.

Figure 6A shows intact granules in the adzuki bean powder. The cell wall formed an encapsulating barrier to adzuki bean powder granules. With the application of Protamax and Viscozyme in the steam-cooked adzuki bean powder, the cell wall was destroyed, causing irreversible damage to the permeability of the cell wall; especially Viscozyme. The Viscozyme used in this study is a complex glycoenzyme with a variety of enzymatic hydrolytic activities, which can effectively degrade cell wall fibrous tissue and increase the release of cellular contents [29]. As can be seen from Figure 6B,C, the binding of amylase to the intracellular starch weakens the blue color of the cell wall and the green color of the starch. In Figure 6C, Viscozyme in-steam cooked adzuki bean powder granules had more cell wall breakage and were more susceptible to enzymatic attacks, which indicated that fiber may have an even greater influence on eGI than protein. Both the soluble and insoluble components exhibited a lower inhibition on enzyme activity compared with their untreated cell counterparts, which could also contribute to the increase in digestion rate [38]. The overall results suggest that the intact cell walls play the following roles: (a) acting as a barrier for the diffusion of the enzyme and (b) providing an adsorptive surface (non-catalytic binding) for alpha-amylase [31,39].

### 3.7. In Vitro Digestion Kinetics and eGI of Deproteined (SC-DP) and Defibered Steamed Cooking Adzuki Bean Powder (SC-DF)

**Figure 7:**
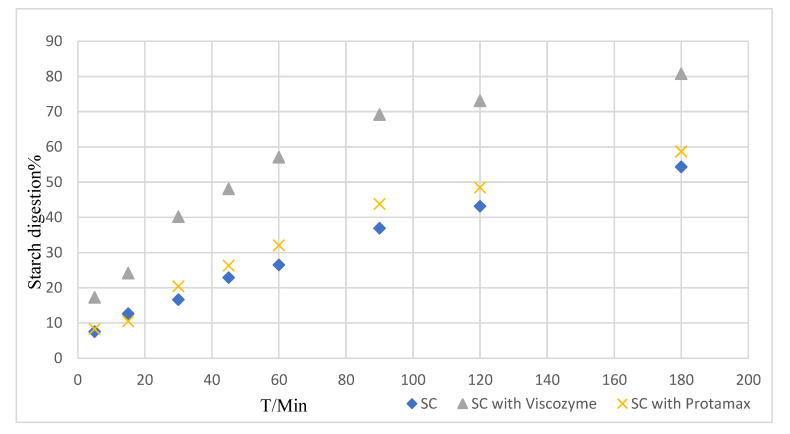
Digestion process of adzuki bean powder prepared by steamed cooking (SC) with different enzyme treatments. SC: steamed cooking adzuki bean powder, SC-DP: steamed cooking with Protamax adzuki bean powder, SC-DF: steamed cooking with Viscozyme adzuki bean powder.

In order to obtain a better understanding of the enzymic digestion mechanism of different gelatinized starch samples, the equilibrium starch hydrolysis percentage (C^∞^) and kinetic constant (k) were estimated by fitting a first-order equation model (Table 3) [40]. The value of k increased from 0.01 min^−1^ for adzuki bean powder to 0.03 min^−1^ for defibered and 0.02 min^−1^ for deproteined adzuki bean powder (Table 3). Compared with steamed cooking the adzuki bean powder, the deproteined and defibered adzuki bean powder could increase the final degree of digestion by 50~200%. The increase of k values indicates that the higher digestion rates were achieved, which means an increasing access of enzymes (Figure 7).

The estimated glycemic index has been used to estimate the blood glucose response after food ingestion by humans [41]. As shown in Table 3, steamed cooking with protease (deprotein) increased the eGI value. This may be because the outer layer of bean starch is wrapped by protein and fiber, making it difficult for amylase to make contact with starch, thus reducing the hydrolysis rate of starch [42,43,44,45]. After steamed cooking with protease (deprotein) and viscozyme (defiber), the membrane on the surface of the adzuki bean powder granules disappeared, the embedding effect of protein on starch granules was weakened, the starch hydrolysis rate increased and the eGI increased. Sciarini et al. [41] also investigated eGI in starchy foods. They believed that the encapsulation of starch within a dietary fiber matrix may delay/prevent their hydrolysis/digestion, leading to lower eGI values. Other researchers have also reached the same conclusion [21,22,23]. In theory, fiber creates a protective layer around the starch granules, limiting the amylase release, and leading to lower viscosity values [21]. This may affect starch digestibility and the glycemic index [41,46]. Similarly, Hardacre et al. [47] found that fibers may exert a non-competitive inhibition on certain enzymes (which may be considered as a chemical barrier), leading to a lower GI value.

Different eGI values could also be attributed to the presence of other components, such as polyphenols in adzuki bean powders, which have been previously described to have lower blood glucose [48]. Future studies could work on the interaction between polyphenols and starch in reducing the eGI in adzuki beans.

## 4. Conclusions

This study investigated the mechanism of different processing methods on the in vitro digestibility and estimated glycemic index (eGI) of adzuki bean powder and the impact of the protein and fiber of steam-cooked adzuki bean powder on the eGI.

By comparing three different processing methods, we found that the eGI value of the steam-cooked adzuki bean powder (SC) was the lowest compared with EC and RC. By observing its microstructure, the SC sample had a better cellular integrity and its cell walls formed a physical barrier to digestive enzymes after cooking. We speculated that steam-cooked processing with wet milling could result in a lower content of damaged starch, meaning that the integrity of starch granules mostly remained and that it is crucial to obtain a low eGI. However, the roller cooking destroyed the cell structure, which led to a relatively completed starch gelatinization, and hence the eGI was higher.

Based on the above results, we utilized two kinds of enzymes (i.e., Protamax and Viscozyme) to prepare deprotein and defiber samples in order to further examine the impact of the enzymatic treatment on the samples’ eGI. Results have confirmed our speculation that protein and fiber impact both the eGI and the in vitro digestion of adzuki bean powder. Additionally, fiber showed the most effect on the eGI compared with protein. The defiber sample’s eGI increased from 49.7 to 83.1 after the enzymatic treatment. It was confirmed that the digestibility of cooked starch in adzuki bean powders was protected by the protein and fiber of the cell wall, leading to a low eGI value. Processing technologies that apply to adzuki bean powder, such as steamed cooking with Viscozyme and Protamax, remove the fiber and protein, which leads to an increase in its in vitro starch digestibility and eGI. We concluded that fiber and protein are crucial to reduce the level of the eGI in adzuki bean powder. We therefore advocate for the development of potential products made from whole adzuki beans in order to maintain its hypoglycemic property.

## Figures and Tables

**Table 1:** In vitro glycemic index of adzuki bean powder prepared by different processing methods.

Processing Methods	K(min^−1^)	C^∞^	eGI
R	0.03	0.91	79.8 ^b^
SC	0.17	0.43	49.7 ^a^
RC	0.03	0.90	80.0 ^b^
EC	0.04	0.60	70.0 ^c^

R: raw adzuki bean powder, SC: steamed cooking adzuki bean powder, RC: roller cooking adzuki bean powder, EC: extruded cooking adzuki bean powder. In the same column of eGI, different superscript letters mean values are significantly different (*p* < 0.05).

**Table 2:** Thermal and crystalline properties of adzuki bean powder prepared by different processing methods.

Processing Methods	To(°C)	Tp(°C)	ΔH(J/g)	Total Crystallinity
R	65.98 ± 0.83 ^b^	73.87 ± 0.16 ^a^	2.91 ± 0.48 ^a^	20.20 ^a^
SC	67.18 ± 1.26 ^a^	69.06 ± 1.24 ^b^	0.03 ± 0.01 ^b^	17.13 ^b^
RC	65.06 ± 1.36 ^b^	68.02 ± 0.40 ^b^	0.01 ± 0.01 ^b^	12.27 ^c^
EC	-	-	-	10.23 ^d^

R: raw adzuki bean powder, SC: steamed cooking adzuki bean powder, RC: roller cooking adzuki bean powder, EC: extruded cooking adzuki bean powder. Different superscript letters within the same column mean values are significantly different (*p* < 0.05).

**Table 3:** Glycemic index of steam-cooked adzuki bean powder prepared with Protamax and Viscozyme.

Sample	K	C^∞^	eGI
SC	0.03	0.70	49.70 ^c^
SC-DP	0.01	0.59	60.50 ^b^
SC-DF	0.02	0.81	83.10 ^a^

SC: steamed cooking adzuki bean powder, SC-DP: steamed cooking with Protamax adzuki bean powder, SC-DF: steamed cooking with Viscozyme adzuki bean powder. In the column of eGI, different superscript letters mean values are significantly different (*p* < 0.05).
